# Metastatic melanoma in an infant: neoadjuvant radiotherapy and radical neck dissection

**DOI:** 10.2340/1651-226X.2026.46049

**Published:** 2026-09-28

**Authors:** Niranjan Bista, Patrick Shenjere, Demeesh Oudit

**Affiliations:** a The Department of Oncoplastic Surgery, The Christie NHS Foundation Trust, Manchester, United Kingdom; b The Department of Histopathology, The Christie NHS Foundation Trust, Manchester, United Kingdom

**Keywords:** melanoma, Sptiz naevus, sentinel lymph node biopsy, radiotherapy, neck dissection

Dear Editor

We report a unique 60-year follow-up of a case that demonstrates the evolution in the classification and management of childhood melanoma. It highlights the long-term melanoma-free survival following neoadjuvant radiotherapy and radical neck dissection at a time when treatment options and diagnostic criteria were limited. The report aims to address the diagnostic and therapeutic challenges of childhood melanoma, highlighting the significant advancement in melanoma care over past six decades.

Melanoma is the 5th most common cancer in the UK [1], with an incidence rate of 16.1 per 100,000 population and a lifetime risk of 1 in 60. In 2008, there were 11,767 new cases [2]. The incidence increases with age, particularly high in males over 50, whereas females under 50 represent 52% of new cases [1]. While melanoma is rare in infants, children, and teenagers, it is one of the most common cancers among those aged 15–34 years [3].

True malignant melanoma in infants and children remains exceptionally rare, accounting for only 0.3–2.0% of all melanomas. Paediatric melanomas pose distinct challenges in diagnosis and treatment, as they lack classical features seen in adult melanomas, complicating primary care diagnosis. The malignancy risk and biological behaviour of Spitz lesions in children are still debated [4, 5].

A congenital melanocytic naevus (CMN) is a benign collection of naevus cells present at birth, occurring in 1% of neonates. Giant CMNs over 20 cm are rare and can be associated with multiple smaller naevi with severe cases covering up to 80% of the body surface area. While the risk of melanoma in CMN is low, the potential of malignant transformation remains clinically significant [6, 7].

Mortality from melanoma in the UK is high, with approximately 2300 deaths annually, translating to over six deaths per day emphasising on effective prevention and early detection [1]. Treatment for melanoma has evolved significantly over the past 60 years, incorporating more conservative surgical methods and new medical therapies.

## Case report

A 54-year-old male Caucasian was diagnosed with malignant melanoma at age 2 years and 9 months, initially diagnosed as a ‘pimple’ and later as an abscess on his right cheek. Following ineffective antibiotic treatment, he underwent incision and drainage, but the lesion reappeared and was diagnosed as pyogenic granuloma. After excision and skin grafting, histopathology revealed incompletely excised juvenile melanoma. He was born healthy, with no prior medical conditions, family history of melanoma, or excessive ultraviolet (UV) exposure.

A month after excision, the patient developed cervical lymphadenopathy and cutaneous nodules on the right cheek, which did not respond to antibiotics. A biopsy of the lymph node confirmed anaplastic melanoma. Following the 1960s practice, radiotherapy was administered to the right cheek and neck, resulting in a slow response.

After a poor response to radiation, the patient had a re-excision of the primary lesion and a right radical neck dissection 6 months later, followed by long-term follow-up. One-year post-surgery, he successfully received treatment for a primary melanoma on his right leg. Recently, he was treated for a Margolin’s ulcer on the right neck scar, later identified as a basal cell carcinoma histologically.

Apart from skin cancer, the patient underwent multiple medical procedures over the years, including the removal of an odontogenic cyst from the upper jaw, a total thyroidectomy due to radiation-induced multi-nodular goitre, removal of a right-sided intracanalicular acoustic neuroma, and nephrectomy for an oncocytoma, with the latter two performed decades after the initial presentation.

At 59 years of age, the patient remains alive, well, and entirely free of recurrence or metastasis from his original melanoma.

Cutaneous melanoma before puberty is rare and categorised into neonatal, prepubescent, and adolescent types, [4] and may arise from giant congenital naevi, transplacental metastases [6, 7], or family traits linked to UV exposure and immunosuppression [4]. Large congenital melanocytic naevi carry a lifetime melanoma risk of approximately 1–2% particularly when associated with neurocutaneous melanocytosis. Early detection is often difficult due to similarities with conditions like Spitz naevus and pyogenic granuloma [8] requiring skilled dermatopathologists to distinguish benign from malignant cases.

In our case, no predisposing factors were identified. Furthermore, maternal transmission appears unlikely and in utero development also seems unlikely as the lesion manifested 2 years post-birth [9–12] suggesting either an unrecognised congenital lesion or sporadic tumour development.

In the past, paediatric melanoma has been difficult to distinguish from Spitz naevi and atypical spitz tumours. In 1948, Spitz described Juvenile Melanoma which follows a benign course. These lesions contain spindled/epithelioid naevomelanocytes with large, irregular cells and abundant eosinophilic cytoplasm, helping to differentiate from malignant melanomas. This clinical behaviour was later reviewed by McWhorter and Woolner at Mayo Clinic in 1954 confirming its benign nature[13].

Spitz tumours exhibit a spectrum from benign Spitz naevi to malignant Spitz melanoma, with atypical Spitz tumours (Spitz melanocytoma) as an intermediate [14, 15]. Atypical Spitz tumours can metastasise regionally, though distant spread is rare. The clinical course of this patient would likely to be a Spitzoid tumour, specifically an atypical Spitz tumour, which shows regional spread without distant metastasis during long-term follow-up.

Recent evidence supports this favourable biological behaviour. Pol-Rodriguez et al. reported an 88% 5-year survival in children younger than 10 years with metastatic spitzoid melanoma, which is significantly better than that observed in older children and adults [16]. Similarly, Roaten et al. found that regional lymph node spread is more common in paediatric melanoma than adult melanoma; however, it is associated with lower recurrence rates [17].

A retrospective Finish study (1990–2014) revealed nearly a four-fold annual increase in melanoma cases among children and adolescents, particularly in spitzoid subtype linked to changing UV exposure habits and a rise in recreational travel, rather than overdiagnosis. The study also found that 66.0% of cases were spitzoid melanoma with 10-year cancer-specific survival of 88.7%. BRAFV600E positivity was associated with poorer prognosis and 8.03% of melanoma deaths. However, sentinel lymph node status did not significantly influence survival, suggesting that regional nodal spread may not carry the same prognostic significance as in adult melanoma [18, 19].

Recent molecular studies have identified alterations involving Cyclin-Dependent Kinase Inhibitor 2A (CDKN2A), B-Raf proto-oncogene, serine/threonine kinase (BRAF), Neuroblastoma RAS Viral Oncogene Homolog (NRAS), KIT Proto-Oncogene, Receptor Tyrosine Kinase (c-KIT), and Telomerase Reverse Transcriptase (TERT) in paediatric melanoma., Although, Spitz tumours more frequently demonstrate Harvey Rat Sarcoma Viral Oncogene Homolog (HRAS) mutations and chromosome 11p amplifications, they are still in a minority of cases [20]. These investigations were however unavailable when this patient was treated and therefore could not contribute to diagnosis or management.

Recent data on paediatric melanoma indicate that positive sentinel lymph nodes increase relapse risk but do not significantly affect overall survival. A 2024 review highlights that while nodal involvement in spitzoid melanoma is common, it may not suggest a poor prognosis. Long-term monitoring is crucial, as some patients can still develop distant metastatic disease [21].

Management of melanoma has changed dramatically over the last 60 years. In the 1960s, standardised excision margins, sentinel lymph node biopsy, cross-sectional imaging, and effective systemic therapies were unavailable. The excision margin for the patient was not recorded, highlighting the absence of established protocols. Prior to surgery, no radiological imaging was completed, and follow-up relied solely on clinical examination and plain X-rays.

Pratt et al. analysed 31 patients under the age of 21 at Christie Hospital with melanoma, out of that 17 had metastatic disease and among them 2 were < 2 years old. Four patients received neo-adjuvant radiotherapy, four received radiotherapy for the treatment of metastases and two patients had chemotherapy [22]. Rao et al. reported on 33 patients aged from 1 day to 20 years between 1967 and 1988, with a median age of 12 years, who underwent therapeutic neck dissections and an elective neck dissection without specifying patient ages [23].

Radiotherapy was frequently used for regional disease, whereas its role today is limited due to the risk of late complications and secondary malignancies in children who are more susceptible to late effects compared to older children [24].

Radical neck dissection in an infant poses significant risks because of the small size of vital neurovascular structures and the potential for lifelong functional morbidity. In this case, despite the presence of significant contracture bands on the right side of the neck, radiation-related complications, including thyroid disease and subsequent basal cell carcinoma, the patient achieved excellent oncological control.

This unique case report details a 2-year-old infant undergoing cervical lymphadenectomy for metastatic melanoma surviving it without further metastases; however, a year later, he developed a second primary tumour and a Basal Cell Carcinoma (BCC) in neck scar tissue which we believe to be a Marjolin’s ulcer. Although melanoma is typically less aggressive in children under 11 years [25], in this case it exhibited aggressive behaviour with regional metastasis to the cervical nodes that was confirmed following excision. The infant underwent a radical neck dissection without prior investigation for distant metastases resulting in a favourable outcome, although such cases are rare today due to the availability of non-surgical treatments.

This case study highlights the historical challenges of treating advanced melanoma in an infant around 60 years ago, emphasising the lack of management guidelines at the time. It notes that despite the risks of long-term tissue morbidity, radical surgical interventions provided definitive cures for regional metastatic disease. This report highlights the significant advancements in melanoma treatment over the past six decades and identifies this case as the youngest patient to undergo radical neck dissection for metastatic melanoma, marking a unique contribution to paediatric melanoma literature.

## Data Availability

The datasets analysed during the current study are not publicly available because they contain identifiable patient information (patient confidentiality) and institutional governance restrictions.
